# Psychosocial outcomes of a non-dieting based positive body image community program for overweight adults: a pilot study

**DOI:** 10.1186/2050-2974-1-44

**Published:** 2013-12-17

**Authors:** Lisa Bloom, Beth Shelton, Melissa Bengough, Leah Brennan

**Affiliations:** 1 School of Health Sciences, RMIT University, Melbourne, Australia; 2 MonashLink Community Health Service, Melbourne, Australia; 3 Southern Health Wellness Recovery Adult Outpatient Eating Disorder Service, Melbourne, Australia; 4 School of Psychology, Australian Catholic University, Melbourne, Australia

**Keywords:** Obesity, Non-dieting, Community, Body image, Intervention, Disordered eating

## Abstract

**Background:**

The limited success of traditional diet focused obesity interventions has led to the development of alternative non-dieting approaches. The current study evaluated the impact of a community based non-dieting positive body image program for overweight/obese people on a range of psychosocial outcomes. The characteristics of this real-world sample presenting for a non-dieting weight management intervention are also described.

**Method:**

Overweight and obese participants enrolled in the eight week ‘No More Diets’ (NMD) group program completed self-report questionnaires assessing disordered eating thoughts and behaviours, body image, motivation for exercise and psychopathology pre- and post-treatment.

**Results:**

Participants (n = 17; 16 female) were aged between 19 and 78 years, with a BMI ranging from 25.2 kg/m^2^ (Overweight) to 55.9 kg/m^2^ (Severely Obese). They reported elevated levels of eating disorder pathology, body shape preoccupation, depression, anxiety and stress compared to community norms (*p* < .05). Following treatment there were significant improvements in reported body shape preoccupation, shape concern and eating attitudes (*p* < .05), and clinically significant changes (small to medium effect sizes; 0.3-0.35) for improvements in reported weight concern, eating competence, stress and health evaluation. There were no changes in reported dietary restraint, emotional eating and uncontrolled eating, or eating concern (*p* > .05).

**Conclusion:**

Individuals presenting for the NMD program demonstrated increased eating disorder pathology and more generalised psychopathology compared to community norms. The NMD program was particularly beneficial for body image and shape concern. Addressing these body image factors may help to address some of the perpetuating factors of obesity and disordered eating, which are often not addressed in the traditional diet-based weight loss interventions.

## Background

Eating disorders, obesity and unhealthy dieting practices share common psychopathology, and can have serious adverse effects on psychological and physical health [1-3], For example, there is evidence that obesity can eventuate in people with bulimic eating disorders and Binge Eating Disorder [4] and levels of body dissatisfaction can be higher in obese populations compared to populations with Anorexia Nervosa [5,6]. Regardless of the method, the goal of traditional obesity interventions, including diet, exercise, behavioural, pharmacological and surgical weight loss, is a reduction in excess body fat, and these approaches often fail to address the perpetuating factors common to both obesity and eating disorders. This emphasis on weight loss is driven by beliefs that excess weight increases morbidity and mortality, sustainable weight loss is possible, and the risks associated with obesity will decrease when weight is lost [7].

The link between obesity and mortality and morbidity is generally accepted [8], however, recent research suggests that this relationship may not be as strong as once thought in the mild to moderately obese [9]. It has also been proposed that some obesity related co-morbidities may be the result of ineffective dieting rather than excess weight [10]. High levels of dietary restraint have been linked to weight gain [11] and to weight cycling [12]. Weight cycling (the repeated gains and losses of weight over time) has been shown to increase risk for cardiovascular disease, depression, and mortality [13-15].

Dietary restraint has also been associated with psychological distress, including anxiety, food and weight preoccupation, disordered eating and eating disorders, particularly binge eating [16-18]. It is believed that dieting creates a vulnerability to binge eating and problems with eating regulation [19]. Research is not clear about what comes first, dieting or binge-eating in obese individuals [20]. Despite this, levels of binge eating and dietary restraint are positively correlated [21] and binge eating, not overall weight, has been found to be a predictor of psychopathology amongst obese treatment-seeking individuals [22]. Additionally, levels of dietary restraint have been found to be comparable between populations with obesity and populations with Anorexia Nervosa [5,19].

Failure to achieve desired weight loss may also lead to further psychological distress [17,18]. Most people enrolling in a weight loss interventions expect to lose up to 30% of their body weight, however short-term weight loss goals of 5-10% are more commonly achieved [21]. A 5-10% weight loss is considered to be clinically significant and is recommended by most expert bodies [23-25]. There are few studies available examining the impact of long-term weight loss on obesity related risk. Those that are available indicate that long-term maintenance of weight loss is rare [26,27] and that the majority of participants are likely to have returned to baseline weight or higher at 2–5 year follow-up [28,29]. There is also increasing evidence that health improvements can occur without significant weight loss in mild to moderately overweight individuals. For example, cardiovascular fitness, blood pressure improvement and reduction in medical symptoms have been shown to occur in response to health behaviour change independently of weight loss [7,30]. Individuals can also improve a number of health outcomes (e.g., recommended physical activity levels, healthy blood pressure, reduced mortality and morbidity rates), whilst still being classified as overweight or obese [31].

Considering that the prevalence of obesity is rising [32], weight loss is difficult to maintain long-term [26,27], and health outcomes can improve in some individuals without weight loss [7,30], alternatives to the traditional obesity treatment paradigm are being explored. Such alternatives include non-dieting programs. Non dieting approaches view weight management from a health-centred approach, with health outcomes as goals rather than weight loss per se. This philosophy is based on the belief that biopsychosocial outcomes can improve without significant weight loss [33,34]. Healthy eating patterns in response to internal satiety and hunger cues, physical activity for the purpose of pleasure and health acceptance of bodies at different shapes and sizes are encouraged [35].

Efficacy studies of non-dieting interventions have demonstrated improvements in eating behaviours and attitudes, including decreases in dietary restraint, disinhibition, overall eating disorder risk, and binge eating, and greater awareness of hunger and satiety cues [5,36-41]. They have also shown physiological changes, including improvements in metabolic fitness (e.g., pulse reading, blood pressure, cholesterol) without significant weight loss [5,36-40]. In a recent review, in four of seven studies, these improvements in the non-dieting group were significantly better than traditional weight control groups [42]. These studies have all examined the efficacy of non-dieting interventions, in controlled efficacy studies [42]. Effectiveness trials (interventions implemented in real-world clinical and community settings) of non-dieting approaches are still required.

The ‘No More Diets’ (NMD) program is an example of a non-dieting community intervention. The program targets those experiencing weight cycling and disordered eating patterns with the goal of achieving a range of health improvements. It targets health behaviours such as; healthy normalised eating patterns based on cues of hunger and satiety, positive body image, and maintained physical movement that is practical, sustainable and enjoyable. The current pilot study will provide an opportunity to examine the effectiveness of the NMD program delivered to overweight and obese adults in a community setting. The current study aimed to examine (1) the psychosocial characteristics of NMD participants, (2) differences in pre-treatment characteristics of participants who did and did not complete the program, and (3) the impact of NMD on psychosocial outcomes of participants.

## Method

### Participants

Participants were recruited to the NMD program via advertisements distributed by MonashLink Community Health Centre, an article in the local newspaper, or through referral from participants’ current health professional at MonashLink. There were no formal exclusion criteria.

### Materials

Participants completed a number of self-report questionnaires:

*Demographic Questionnaire* This questionnaire was developed for the purpose of the NMD program. It includes information about height and weight, family structure, educational level, employment status, eating and activity history.

*Eating Disorder Examination - Questionnaire* (EDE-Q) [43]. This 28-item questionnaire measures four aspects of the psychopathology of eating disorders. The items are rated on a 6-point scale with higher scores reflecting greater severity of psychopathology. The four subscales are Restraint (e.g., avoidance of food, dietary rules), Eating Concern (e.g., food preoccupation), Weight Concern and Shape Concern (e.g., preoccupation and dissatisfaction with weight and shape). It has adequate internal consistency (α = .70, .73, .72 and .83 respectively) and convergent and discriminant validity (*r* = .79-.81 and .78-.85) [43,44].

*Three Factor Eating Questionnaire* - *Revised 21 Items* (TFEQ-21) [45]. This revised version of the Three Factor Eating Questionnaire measures eating behaviour in three domains [46]. Twenty-one items comprise the revised scale; Cognitive Restraint (restricting food intake in order to influence weight), Emotional Eating (eating in response to negative mood states) and Uncontrolled Eating (overeating behaviours). Items are rated on a 4-point scale. The scales have adequate internal consistency (α = .76, .83 and .85 respectively) and convergent and discriminant validity [45].

*ecSatter Inventory*[47]. This 16-item scale measures eating competence. Items are rated on a 5-point Likert scale. The four subscales are Contextual Skills (measuring skills relate to selecting, preparing and planning meals), Eating Attitude (measuring levels of comfort associated with food and eating), Food Acceptance (openness to a wide range of foods) and Internal Regulation (eating behaviours in response to internal satiety cues), and a total Eating Competence score. It has adequate construct validity and internal consistency (α = .79, .82, .70, .71 and .85 respectively) [47,48].

*Multidimensional Body Self-Relations Questionnaire* (MBSRQ) [49]. This 69-item questionnaire measures body image and attitudes towards the self. The Appearance Evaluation (measuring self-ratings and attitudes towards one’s appearance), Fitness Evaluation (self-ratings and attitudes towards fitness levels) and Health Evaluation (self-ratings of feelings of healthiness) subscales were used for the current study. Items are rated on a 5-point scale ranging from ‘definitely disagree’ to ‘definitely agree’. The scales have adequate internal consistency (α = .88, .77 and .83 respectively) [49].

*Body Shape Questionnaire* (*BSQ*) [50]. This 36-item scale measures body shape preoccupations and levels of body dissatisfaction and associated distress over a 4 week period. Items are rated on a 6-point Likert scale ranging from ‘Never’ to ‘Always’. It has adequate reliability (α = .88) and good concurrent validity for clinical and non-clinical samples [51].

*Depression*, *Stress and Anxiety Scale* (DASS) [52]. This 42-item questionnaire measures symptoms of depression, anxiety and stress. The three subscales (Depression, Anxiety and Stress) are answered on a 4-point severity/frequency scale of symptoms over the last week. It has adequate internal consistency (α = .81, .73 and .81 respectively) [42] and convergent and discriminant validity [53].

*Exercise Motivations Inventory* - *version 2* (EMI-2) [54]. This 51-item questionnaire measures reasons for exercise. The subscales used in the current study were Enjoyment and Weight Management (e.g., Personally I exercise, or might exercise to stay slim). Items are rated on a 5-point scale. It has adequate reliability (α = .89 and .91) and convergent and discriminant validity for both exercisers and non-exercisers [54].

### Procedure

This study was approved by the Monash University Human Research Ethics Committee and the RMIT University Human Research Ethics committee prior to commencement. The program was delivered at MonashLink Community Health Service, a non-profit organization funded by a variety of government programs, providing a range of health and well-being services to people living and working within the City of Monash, Melbourne, Victoria, Australia. Participants approached the community centre for the purposes of attending the NMD program.

A psychologist (BS) and a dietician (MB) from MonashLink Community Health’s Disordered Eating Service adapted the NMD program from the Set Your Body Free programs of Paxton and colleagues [55-57]. The program consisted of 8 weekly 2 hour sessions; session content is outlined in Table 1. The program ran twice over a 6 month period. The first program was facilitated by a psychologist and dietician, and the second by a dietician and mental health counsellor. The initial group had 12 participants; the second group had 9 participants.

**Table 1:** NMD weekly session outline

**Week**	**Food & eating topics**	**Body image topics**	**Movement activity**
1	Why diets do not work	Looking at spine and identifying basic spinal movements to music	
2	Regular eating pattern	Exploration of pros and cons of body acceptance and body dissatisfaction	Introduction to image-based movement improvisation
3	Hunger Scale	Introduction of concept of overvaluation of shape & weight	Integration of a learnt sequence in pairs and improvisation
4	Hungry eating vs non-hungry eating	Introduction of concepts of extreme &/or unhelpful cognitions regarding body image	Continued integration of learnt sequences and reflection of effects of movement
5	How to reduce non-hungry eating	Coping with extreme &/or unhelpful cognitions regarding body image	Movement composition to music
6	Eating with awareness	Review of concepts & effects of overvaluation of shape & weight, avoidance & body checking	Movement sequences and improvisation with reflection
7	Fine tuning nutritional knowledge	Linking movement with community options: guest instructor to demonstrate available exercise programs	
8	Overview and reflection		

Note: Each session ran for 2 hours.

In the first week of the program, participants completed the questionnaire booklet as part of the program evaluation. A plain language statement and consent form invited the participants to allow the data in the questionnaires to be used in the current study. During the final session, all participants were given the questionnaire booklet to complete after the program and post back to the NMD facilitators. Missing data was collected via telephone contact with participants following the return of their questionnaires. The researcher was provided with the data of those participants who had consented for their data to be used in research.

### Statistical analyses

All statistical analyses were completed using the software package Statistical Package for Social Sciences (SPSS; Version 20). Data screening, missing data analysis and assumption testing were conducted prior to commencement of data analysis. There was no missing data. As this was a study of a ‘real-world’ intervention taking place in the community, power analysis was not completed. Similarly, as this was an exploratory study alpha levels were not adjusted to account for inflation in Type 1 error due to multiple comparisons. Given the small sample size and the exploratory nature of this study both statistically significant and clinically significant results are discussed. Effect sizes are also presented using the following range: *r* = .2 small, *r* = .5 medium, and *r* = .8 large effect size [58]. Non-parametric tests were used due to the small sample size. In line with the requirements of non-parametric tests, medians are reported instead of means for analyses where appropriate.

The psychosocial characteristics of the sample were described by comparing scores on the measures to published norms from normal and clinical samples using a Wilcoxon Signed Rank test. Normative comparative samples were selected on the basis that they used the appropriate measures, and participant characteristics (e.g., sex) could be matched where possible. The EDE-Q normal sample consisted of females aged over 16 years [59]. The TFEQ sample consisted of middle-aged females [60]. The EcSatter sample consisted of overweight and physically active adults [47]. The MBSRQ sample consisted of females aged over 18 years [49]. The BSQ normal sample consisted of female university students [50] and the DASS 21 normal sample consisted of males and females aged over 17 years [52]. For clinical samples, a diagnosis of an eating disorder and the relevant measure was required, as well as matching characteristics where possible. The EDE-Q clinical sample consisted of females with Anorexia Nervosa [59]. The BSQ sample consisted of females with Bulimia Nervosa [50]. The DASS 21 sample consisted of adolescents and adults with Anorexia Nervosa [61]. The sample was also compared to clinical cut-offs where available. Participants who completed the program were compared with data from participants who dropped out using a Mann–Whitney test. Pre- and post-scores on the outcome measures were compared using related samples Wilcoxon Signed Rank tests.

## Results

**Figure 1:**
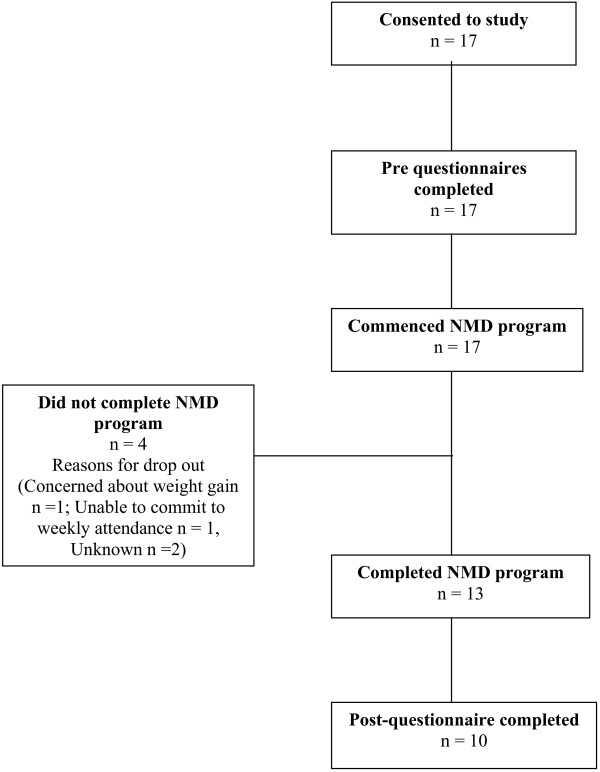
**Data collection process from consent stage to return of post-questionnaire.**

Of the 21 participants who enrolled in the NMD program, 17 (81%) consented to participate in the study, as outlined in Figure 1. The participants (16 females, 1 male) were aged between 19 and 78 years (*M* = 56.45, *SD* = 16.19). The majority were born in Australia (71%) and 82% identified Australian as their ethnicity. Thirty-five per cent were married, 6% defacto, 18% divorced or separated, 18% never married, and 23% widowers. Almost half (47.1%) had trade or tertiary qualifications. Twenty-four percent were employed (12% part-time; 12% full-time), 35% were retired and 41% were either unemployed, unable to work, or not working due to caring/parenting commitments. Participants’ self-reported weight ranged between 69.8 to 156.0 kg (*M* = 92.69, *SD* = 20.88) and their body mass index (BMI; kg/m^2^), calculated based on self-reported height and weight, ranged between 25.2 (Overweight) to 55.9 (Severely Obese) (*M* = 34.17, *SD* = 8.57).

Table 2 illustrates the means and standard deviations for each variable, and clinical-cut offs where appropriate. Over a third (35.4%) of participants scored within the clinical range for concerns about weight. More than half (59%) the participants scored within the clinical range on levels of depression, while almost half scored within the clinical range on levels of stress (41%) and anxiety (47%). Participants reported that binge eating episodes occurred on a mean of 4.65 days (*SD* = 6.23) in the past 28 days.

**Table 2:** **Descriptive statistics and clinical cut**-**offs for all variables**

**Variable**	***M***	***SD***	**Range**	**Clinical cut-off score**	**% in clinical range**
Self-report weight (kg)	92.69	20.88	69.8 – 156.0	N/A	N/A
Body Mass Index	34.17	8.57	25.2 - 55.9	Above 25 = Overweight, Above 30 = Obese	29.470.6
*Eating Disorders Examination* - *Questionnaire*					
Binge Eating Frequency (per 28 days)	4.65	6.24	0- 23		
Restraint	1.51	0.94	0 - 6	≥ 4	0
Eating Concern	1.71	1.44	0 - 6	≥ 4	11.8
Shape Concern	3.67	1.63	0 - 6	≥ 4	59.0
Weight Concern	3.24	1.50	0 - 6	≥ 4	35.4
*Three Factor Eating Questionnaire* - *Revised*					
Cognitive Restraint	2.50	0.68	0 - 4	N/A. Higher scores indicate higher cognitive restraint.	N/A
Emotional Eating	2.76	0.89	0 - 4	N/A. Higher scores indicate higher levels of emotional eating.	N/A
Uncontrolled Eating	2.56	0.52	0 - 4	N/A. Higher scores indicate higher levels of uncontrolled eating and impulsivity.	N/A
*ecSatter Inventory*					
Eating Attitudes	8.94	3.88	0 - 15	N/A. Higher scores indicate more relaxed and open attitudes to eating	N/A
Food Acceptance	4.71	2.37	0 - 9	N/A. Higher scores indicate higher levels of cognitive and behavioural food acceptance.	N/A
Internal Regulation	6.47	2.79	0 - 9	N/A. Higher scores indicate stronger endorsement of the experiential processes of hunger, appetite and satiety.	N/A
Contextual Eating Skills	8.35	3.71	0 - 15	N/A. Higher scores indicate more behaviours related to managing the patterns and opportunities for eating and planning meals.	N/A
Eating Competence	28.47	10.94	0 - 48	≥ 32 = Eating competent	47.1
*Multidimensional Body Self- Relations Questionnaire*					
Appearance Evaluation	2.18	0.98	0 - 5	N/A. High scores indicate more positive feelings about appearance.	N/A
Fitness Evaluation	2.94	0.95	0 - 5	N/A. Higher scores indicate greater feelings of fitness.	N/A
Health Evaluation	3.14	0.84	0 - 5	N/A. Higher scores indicate greater feelings of being in good health.	N/A
*Body Shape Questionnaire*	106.29	33.20	34 - 204	N/A. Higher scores indicate greater concern and preoccupation with appearance and shape.	N/A
*DASS*-*21*					
Depression	14.47	10.04	0 - 42	N/A. Moderate, Severe and Extremely Severe range.	59.0
Anxiety	9.41	6.24	0 - 42	N/A. Moderate, Severe and Extremely Severe range.	47.1
Stress	17.06	10.47	0 - 42	N/A. Moderate, Severe and Extremely Severe range.	41.3
*Exercise Motivations Inventory* - *2*					
Reasons for exercise: Enjoyment	2.01	1.82	0 - 5	N/A. Higher scores indicate more endorsement of enjoyment as a motivator for exercise.	N/A
Reasons for exercise: Weight management	3.69	1.21	0 - 5	N/A. Higher scores indicate more endorsement of weight management as a motivator for exercise.	N/A

Note: The clinical range for the DASS-21 consists of scores in the Moderate range and above.

Scale scores obtained from the study sample were compared to the most appropriate community and clinical samples (Table 3). Severity of eating disorder pathology was compared to a normal [43] and a clinical eating disorder female sample [59]. Participants reported significantly higher scores compared to the community sample on levels of eating concern, shape concern and weight concern. Participants obtained lower scores compared to a sample of females with Anorexia Nervosa on levels of restraint, eating concern, and weight concern.

**Table 3:** **Results of Wilcoxon Signed Rank test comparing the pre**-**test study sample to normal and clinical samples**

**Variable**	**Study sample (n** **=** **17)**	**Normal sample**		**Clinical sample**	
	***Mdn *** **( *Interquartile range *; *IQR*)**	***M *(*SD***)	**Wilcoxin *T *** **& effect size ( *r *)**	***M *** **( *SD *)**	**Wilcoxin *T *** **& effect size ( *r *)**
*Eating Disorders Examination* - *Questionnaire*		(n = 243)^a^		(n = 60)^b^	
Restraint	1.2 (1.4)	1.3 (1.3)	*T* = 93, *p* = .434, *r* = .19	4.7 (1.6)	*T* < .01, *p* < .001, *r* = -.88
Eating Concern	1.4 (2.1)	0.6 (0.9)	*T* = 130, *p* = .011, *r* = .61	4 (1.3)	*T* = 2, *p* < .001, *r* = -.83
Shape Concern	4.4 (3.0)	2.2 (1.6)	*T* = 138, *p* = ,004, *r* = .71	4.6 (1.4)	*T* = 39, *p* = .076, *r* = .78
Weight Concern	3.4 (2.8)	1.6 (1.4)	*T* = 141, *p* = .002, *r* = .74	4.8 (1.2)	*T* = 9, *p* = .001, *r* = -.78
*Three Factor Eating Questionnaire* - *Revised*		(n = 284)^c^			
Cognitive Restraint	2.5 (1.2)	1. 6 (0.8)	*T* = 150, *p* < .001, *r* = .85	N/A	N/A
Emotional Eating	2.8 (1. 3)	1.7 (1.2)	*T* = 146, *p* = .001, *r* = .80	N/A	N/A
Uncontrolled Eating	2.77 (.8)	1.1 (0.8)	*T* = 153, *p* < .001, *r* = .88	N/A	N/A
*ecSatter Inventory*		(n = 832)^d^			
Eating Attitudes	9.0 (4.0)	11.1 (3.0)	*T* = 45, *p* = .135, *r* = -.36	N/A	N/A
Food Acceptance	6.0 (3.5)	4.9 (2.2)	*T* = 73, *p* = .868, *r* = -.04	N/A	N/A
Internal Regulation	7.0 (3.5)	6.7 (1.8)	*T* = 85, *p* = .686, *r* = .01	N/A	N/A
Contextual Eating Skills	9.0 (4.5)	8.5 (3.3)	*T* = 76, *p* = .981, *r* = -.01	N/A	N/A
Eating Competence	29.0 (13.5)	31.1 (7.5)	*T* = 59, *p* = .407, *r* = -.20	N/A	N/A
*Multidimensional Body Self-Relations Questionnaire*		(n = 1070)^e^			
Appearance Evaluation	2.0 (1.1)	3.4 (0.9)	*T* = 7, *p* = .001, *r* = -.80	N/A	N/A
Fitness Evaluation	2. 7 (1. 7)	3.5 (1.0)	*T* = 36, *p* = .055, *r* = -.47	N/A	N/A
Health Evaluation	3.7 (1.2)	3.9 (0.8)	*T* = 15, *p* = .004, *r* = -.71	N/A	N/A
		(n = 535)^f^		(n = 38)^g^	
*Body Shape Questionnaire*	112.0 (57.5)	81.5 (28.4)	*T* = 128, *p* = .015, *r* = .59	136.9 (22.5)	*T* = 17, *p* = .005, *r* = -.68
*DASS*-*21*		(n = 592)^h^		(n = 27)^i^	
Depression	14.0 (18.0)	6.3 (7.0)	*T* = 132, *p* = .009, *r* = .64	21.6 (10.7)	*T* = 28, *p* = .022, *r* = -.58
Anxiety	18.0 (10.0)	4.7 (4.9)	*T* = 131, *p* = .010, *r* = .63	16.2 (9.1)	*T* = 8, *p* = .001, *r* = -.79
Stress	16.0 (17.0)	10.1 (7.9)	*T* = 122, *p* = .031, *r* = .52	24.8 (9.3)	*T* = 26, *p* = .017, *r* = -.58

Note: *N*/*A*: analysis not completed as no appropriate comparable sample available.

^a^females over 16 years [59]. ^b^females with Anorexia Nervosa [59].^c^middle-aged females [60]. ^d^overweight and physically active adults [47]. ^e^females over 18 years [49]. ^f^female university students and family planning clinic attendees [50]. ^g^females with Bulimia Nervosa [50]. ^h^males and females, aged over 17 years, in the community [52]. ^i^adolescents and adults with Anorexia Nervosa [61].

Level of preoccupations and concerns about body image were also compared to a community sample of women and a clinical sample of women with Bulimia Nervosa [50]. Participants scored significantly higher on the BSQ than the community sample, and lower than the clinical sample. Compared to a normal sample of adult females [52], participants scored significantly higher on measures of depression, anxiety and stress. Participants scored significantly lower compared to an Australian sample of females with Anorexia Nervosa [61].

Eating behaviour was compared to a normal sample of middle-aged adults [60]. Participants obtained significantly higher scores compared to the normal sample on levels of eating restraint, emotional eating, and uncontrolled eating. Attitudes towards the self and body image scales were compared to a normal sample of female adults aged over 18 years [49]. Participants obtained significantly lower scores on the Appearance Evaluation subscale (feelings of attractiveness) and the Health Evaluation subscale (feelings of health).

Table 4 illustrates the results examining predictive characteristics of treatment retention and outcome. At pre-intervention, the level of endorsement of weight management as a motivator for exercise was the only statistically significant difference between NMD completers and non-completers. Completers endorsed weight management for exercise significantly more than non-completers. Small to medium effect sizes were observed for differences between completers and non-completers on shape concern, cognitive restraint and health evaluation; completers scoring higher than non-completers on these subscales.

**Table 4:** **Results of Mann**–**Whitney tests domparing completers and non**-**completers for all variables**

**Variable**	**Completion n** **=** **13 *Mdn *( *IQR *)**	**Non-completion n** **=** **4 *Mdn *( *IQR *)**	**Mann–Whitney *U *** **&effect size ( *r *)**
Self - reported weight (kg)	91 (28.3)	85 (56.5)	*U* = 27, *p* = 1.00, *r* = -.03
Body Mass Index	32 (8.7)	31.55 (23.6)	*U* = 26, *p* = 1.00, *r* < .01
*Eating Disorders Examination* - *Questionnaire*			
Restraint	1.4 (1.5)	1.1 (1.4)	*U* = 21.5, *p* = .624, *r* = -0.12
Eating Concern	1.4 (2.7)	1.2 (2.0)	*U* = 18, *p* = .412, *r* = -0.22
Shape Concern	4.4 (2.1)	2.4 (3.4)	*U* = 12, *p* = .130, *r* = -0.39
Weight Concern	3.4 (1.9)	2.4 (3.4)	*U* = 17.5, *p* = .350, *r* = -0.23
*Three Factor Eating Questionnaire* - *Revised*			
Cognitive Restraint	2.9 (1.2)	2.2 (1.0)	*U* = 14.5, *p* = .201, *r* = -0.32
Emotional Eating	2.8 (1.6)	2.4 (1.2)	*U* = 20.5, *p* = .549, *r* = -0.15
Uncontrolled Eating	2.33 (0.9)	2.8 (0.9)	*U* = 18.5, *p* = .412, *r* = -0.21
*ecSatter Inventory*			
Eating Attitudes	9 (5.0)	10 (8.8)	*U* = 25, *p* = 1.00, *r* = -0.03
Food Acceptance	6 (4.5)	4 (2.5)	*U* = 20, *p* = .549, *r* = -0.17
Internal Regulation	7 (3.5)	7 (6.8)	*U* = 23, *p* = .785, *r* = -0.08
Contextual Eating Skills	9 (5.0)	7 (5.5)	*U* = 17, *p* = .350, *r* = -0.25
Eating Competence	29 (18.0)	28.5 (22.5)	*U* = 21, *p* = .624, *r* = -0.14
*Multidimensional Body Self-Relations Questionnaire*			
Appearance Evaluation	2.0 (0.71)	2.6 (2.0)	*U* = 22.5, *p* = .703, *r* = -0.1
Fitness Evaluation	2. 7 (1.8)	2.67 (1.4)	*U* = 19, *p* = .477, *r* = -0.19
Health Evaluation	3.1 (1.4)	2.8 (1.1)	*U* = 14, *p* = .202, *r* -0.33
*Body Shape Questionnaire*	112.0 (42.0)	104.0 (92.5)	*U* = 25.5, *p* = .956, *r* = -0.01
*DASS* - *21*			
Depression	14 (17.0)	9 (24.5)	*U* = 20, *p* = .549, *r* = -0.17
Anxiety	8 (10.0)	10 (18.5)	*U* = 23.5, *p* = .785, *r* = -0.07
Stress	16 (15.0)	20 (27)	*U* = 20.5, *p* = .549, *r* = -0.15
*Exercise Motivations Inventory* - *2*			
Reasons for exercise: Enjoyment	1.5 (3.6)	1.1 (2. 6)	*U* = 18.5, *p* = .412, *r* = -0.21
Reasons for exercise: Weight management	4.5 (1.4)	2.8 (2.4)	*U* = 5.5, *p* = .015, *r* = -0.57

Table 5 illustrates results of statistical testing for differences between pre- and post-intervention variables. There were statistically significant improvements in scores on shape concern and attitudes towards eating. Concerns and preoccupation with the body image also significantly decreased from pre- to post-intervention. Small to moderate effect sizes were observed for improvements in levels of restraint, weight concern, stress, food acceptance, and the health evaluation and fitness evaluation subscales.

**Table 5:** **Results of related samples Wilcoxon Signed Ranks test for differences between pre**- **and post**-**intervention for all variables**

**Variables**	**Pre *Mdn *** **( *IQR *)**	**Post *Mdn *** **( *IQR *)**	**Wilcoxon T and effect size ( *r *)**
Self - reported weight (kg)	90.5 (23.3)	90.0 (25.7)	*T* = 21, *p* = .507, *r* = -.16
Body Mass Index	31.2 (8.6)	31.8 (7.2)	*T* = 23, *p* = .646, *r* = -.11
*Eating Disorders Examination* - *Questionnaire*			
Binge Eating Frequency (per 28 days)	2.0 (6.2)	0.0 (3.6)	T = 15 , *p* = .176, *r* = -.33
Restraint	1.2 *(*1.4*)*	0.6 (2.3)	*T* = 15, *p* = .202, *r* = -.31
Eating Concern	1.4 (2.1)	0.5 (2)	*T* = 10.5, *p* = .153, *r* = -.35
Shape Concern	4.4 (3)	2.3 (2.8)	*T* = 0, *p* = .005, *r* = -.68
Weight Concern	3.4 (2.8)	2.8 (3.2)	*T* = 9.5, *p* = .066, *r* = -.44
*Three Factor Eating Questionnaire* - *Revised*			
Cognitive Restraint	2.5 (1.2)	2.5 (0.6)	*T* = 16, *p* = .439, *r* = -.19
Emotional Eating	2.8 (1.3)	2.5 (2.0)	*T* = 23, *p* = .646, *r* = -.11
Uncontrolled Eating	2.7 (0.8)	2.4 (1.5)	*T* = 29.5, *p* = .838, *r* = -.05
*ecSatter Inventory*			
Eating Attitudes	9.0 (4)	11.5 (6)	*T* = 33.5, *p* = .028, *r* = -.53
Food Acceptance	6.0 (3.5)	7.0 (2)	*T* = 13, *p* = .131, *r* = -.37
Internal Regulation	7.0 (3.5)	7.0 (3)	*T* = 11, *p* = .334, *r* = -.23
Contextual Eating Skills	9.0 (4.5)	9.5 (6.3)	*T* = 20.5, *p* = .725, *r* = -.09
Eating Competence	29.0 (13.5)	36.5 (19.3)	*T* = 36, *p* = .108, *r* = -.39
*Multidimensional Body Self-Relations Questionnaire*			
Appearance Evaluation	2.0 (1.1)	2.4 (1.3)	*T* = 39.5, *p* = .220, *r* = -.30
Fitness Evaluation	2.7 (1.7)	4.2 (1.6)	*T* = 22, *p* = .161, *r* = -.34
Health Evaluation	3.0 (1.2)	3.5 (1.5)	*T* = 36.5, *p* = .095, *r* = -.40
*Body Shape Questionnaire*	112.0 (57.5)	89.0 (73.5)	*T* = 1, *p* = .007, *r* = -.66
*DASS*-*21*			
Depression	14 (18.0)	5 (15.0)	*T* = 15.5, *p* = .398, *r* = -.20
Anxiety	8 (10.0)	3 (8.5)	*T* = 13.5, *p* = .284, *r* = -.26
Stress	16 (17.0)	10 (14.5)	*T* = 7, *p* = .121, *r* = -.38
*Exercise Motivations Inventory* - *2*			
Reasons for exercise: Enjoyment	1.5 (3.4)	3.4 (4.4)	*T* = 18, *p* = .490, *r* = -.17
Reasons for exercise: Weight management	4 (1.4)	3.9 (2.3)	*T* = 6, *p* = .172, *r* = -.33

## Discussion

The study aimed to describe the characteristics of participants in the community attending the NMD program and to investigate characteristics that were predictors of treatment retention. The primary aim of the current study was to evaluate the NMD program on a range of health outcomes in an uncontrolled pilot study.

Compared to community samples, participants enrolled in the NMD reported elevated levels on all eating behaviour and psychosocial outcomes, except for subscales of the ecSatter Inventory, the restraint scale from the EDE-Q, and subjective feelings of fitness (Fitness Evaluation subscale), although a medium to large effect size for this difference (*r* = -.47) was observed. These findings are consistent with previous research that has indicated that although obese individuals do not exhibit a greater level of psychopathology than the normal population, treatment-seeking individuals are more likely to exhibit a greater level of psychological disturbance and disordered eating patterns [8,62,63]. Compared to clinical eating disorder samples, participants scored lower on eating disorder pathology (restraint, weight and eating concern), depression, anxiety, stress and body preoccupation. NMD participants had similar levels of shape concern to the clinical sample, highlighting the need for obesity treatment to tackle body image problems.

Pre-treatment characteristics were generally not predictive of treatment retention or treatment outcomes. The exception was reasons for exercise; completers endorsed weight management as a motivator for exercise significantly more than non-completers. There were small to medium effect sizes for differences between completers and non-completers on levels of shape concern, cognitive restraint and health evaluation, with a trend for completers to have greater shape concern, higher cognitive restraint and higher ratings of feelings of healthiness. These findings are consistent with a recent review of predictors of attrition in obesity interventions, which found that while there were no consistent predictors of treatment attrition, there were several factors associated with attrition, including greater body dissatisfaction, more dieting attempts and poor mental health [64]. Lack of significant predictors is likely due, at least in part, to the small sample size.

Following the completion of the NMD program, there were improvements on several biopsychosocial health outcomes. There was a significant increase in positive attitudes towards food and eating, and a decrease in body shape preoccupation, dissatisfaction and shape concern. This finding is consistent with previous research [5,6,38,65-67]. For example, Tanco and colleagues found that a non-dieting intervention resulted in improvements in eating related psychopathology compared to a dieting intervention despite similar weight loss. Non-dieting interventions, such as NMD may be particularly beneficial for treating the psychological correlates of obesity including body image dissatisfaction.

The results for improvements in restraint, weight concern, stress, food acceptance, and the health evaluation and fitness evaluation subscales were not statistically significant, however small to medium effect sizes were observed. These results suggest there have been clinically significant improvements which were not statistically significant as a result of the small sample size. There was also a small to medium effect for improvements in overall eating competence (which incorporates the factors of eating attitudes, food acceptance, internal regulation and contextual eating skills), which suggest there may have been some clinically meaningful change in eating attitudes. Descriptively, the pre-treatment median (29) falls outside of the Eating Competent range, whereas the post-treatment median (36.5) falls within the Eating Competent range, suggesting a trend for a meaningful improvement in eating competence.

Despite changes in these eating and body image factors, there were no differences post-intervention on levels of dietary restraint, emotional eating and uncontrolled eating, or eating concern [43]. Given that non-dieting approaches encourage intuitive eating and normalised eating as a sustainable approach to healthy eating, rather than a short-term weight loss solution [10], and that level of restraint was elevated in the current sample, it would be expected there would be improvements in measures of eating behaviours and attitudes in the current NMD intervention. Studies have shown improvements in disordered eating post-intervention [5,6,37-39]. The programs from these studies ranged in length from 10 to 24 sessions, with an average length of 14 sessions. The current NMD program ran for 8 weeks, and it may be that eating behaviours (restraint, uncontrolled eating and emotional eating) are more resistant to change, and therefore require a longer time period (more sessions) or more targeted intervention for changes to achieve significant improvements in eating behaviours. The degree of endorsement of weight management and enjoyment as motivators for exercise also did not change following the intervention. As people who seek treatment are likely to be more motivated than non-treatment seekers, this may account for why the NMD participants’ positive endorsement of exercise did not change post-intervention.

Small (non significant) effects were observed for changes in levels of depression and anxiety, suggesting there was little improvement following the intervention. As the NMD program had no exclusion criteria, it is possible that some participants were experiencing co-morbid disorders. The baseline characteristics are consistent with this explanation, with more 50% of participants scoring within the clinical range of depression, and over 40% within the clinical range for anxiety and stress. This is consistent with previous research indicating elevated levels of depression and anxiety in obese treatment seekers. The presence of co-morbid mood disorders and mood regulation difficulties was not a target of treatment. These would be common phenomena in real-world obesity interventions that may not be reflected in efficacy studies, as participants with co-morbid disorders are typically excluded from such studies.

Weight and BMI was maintained post-intervention. This was expected as weight loss was not a goal of the current intervention. The non-dieting approach posits that weight loss is not required for health improvements (unless at extreme ends of the spectrum) and this weight maintenance is similar to previous findings in non-dieting interventions [36,38,39,66]. Some healthcare professionals fear that this approach will lead to weight gain [38], however, the current results add to previous research demonstrating an absence of significant weight gain following non-dieting interventions [10]. The frequency of binge episodes did not decrease significantly, but there was a trend for a reduction in the frequency post-intervention. Bacon and Aphramor’s review evaluates the evidence and rationale for a shift from the traditional weight-focused treatment paradigm, which induces only short-term weight loss, and little benefits of improved morbidity and mortality. Together, the results of the current study add further support for a paradigm shift in the treatment of overweight and obesity [10].

This study has a number of strengths. The major strength is that it is an effectiveness study with high external validity. These participants are representative of treatment-seeking individuals from the community, accepted without exclusion criteria unlike some other studies of non-dieting interventions. In comparison, with the goal of achieving high internal validity, efficacy trials typically require participants to meet strict inclusion criteria, often excluding those with common mental health conditions and/or or medical complications. In comparison to some other non-dieting interventions, the NMD program also has a large focus on movement that is practised in session. This movement does not require specialist skills, as opposed to exercise or moderate physical activity promoted in other studies. The movements were demonstrated in session, and were aimed at people of all ages, all sizes, and those with limited mobility. Furthermore, much of the previous research into obesity treatment focuses on medical and surgical interventions or traditional diet-based weight loss programs; this is one of few evaluations of non-dieting approaches. This study examines an intervention that is easily accessible, easily delivered within the community, and may potentially be suited to a wider range of treatment-seekers.

A number of limitations should be considered when interpreting the results of the current study. Firstly, as this is an effectiveness study without a control group, internal validity is limited. In addition, as the sample size is small the conclusions drawn from the statistical analyses should be interpreted with caution, as a larger sample size is required to confirm results, and determine if results are able to be generalised to the population. Additionally, the impact of self-selection bias cannot be ruled out. Participants that selected into the study may be different to participants who completed the NMD program but did not elect to take part in the current study, and this needs to be considered when interpreting the results.

A significant limitation which is common to other studies of non-dieting interventions is that physiological health indicators (e.g., cholesterol, blood pressure, metabolic rates) were not included in the outcome measures. Evidence of changes in physiological outcomes are important in order to understand the role of physiological health improvements independent of significant weight loss and should be assessed in future non-dieting interventions.

The limitations could be addressed in future research by conducting larger-scale studies with larger sample sizes within the community. Follow-up data would also provide useful information. In addition, as obesity is associated with a number of physical, psychological and behavioural profiles, in order to understand the effectiveness and sustainability of alternative obesity treatments evaluations of future interventions should incorporate physiological, psychological and behavioural (including eating and exercise behaviours) outcome measures. This will add to the current understanding of obesity and associated characteristics, and how to best target these factors in treatment.

## Conclusion

The current study provides preliminary evidence of the effectiveness of the NMD program for improvements in body shape preoccupation and dissatisfaction and attitudes towards eating. The study has also highlighted the physical, psychosocial and behavioural characteristics of a treatment-seeking overweight/obese sample in a non-dieting based community program. As obesity has been associated with several disordered eating maintaining factors (binge eating, emotional eating, body image dissatisfaction, weight cycling, disordered eating [10]), these factors need to be incorporated as part of treatment. They are not addressed entirely through weight loss focused interventions, and alternative approaches, such as the NMD program, may be a viable alternative treatment paradigm which focus on health outcomes, including normalised eating, body self-acceptance and sustainable and realistic physical activity.

## Competing interests

The authors declare that they have no competing interests.

## Authors’ contributions

LB, a research student, contributed to the design of the study, was responsible for data collection, analysis and interpretation and lead the manuscript development. BS and MB adapted and delivered the intervention and provided feedback on study design, measure selection and manuscript development. LB supervised LB and was responsible for the design of the study, measure selection, planning of statistical analyses, and overseeing manuscript development. All authors read and approved the final manuscript.

## References

[B1] Fairburn CG Cognitive Behavior Therapy and Eating Disorders 2008 New York: The Guilford Press

[B2] Neumark-Sztainer D Wall M Guo J Story M Haines J Eisenberg M Obesity, disordered eating, and eating disorders in a longitudinal study of adolescents: How do dieters fare 5 years later? J Am Diet Assoc 2006 1 4 559 568 10.1016/j.jada.2006.01.003 16567152

[B3] Fairburn CG Brownell KD Eating Disorders and Obesity 2002 2 New York: The Guilford Press

[B4] Fairburn C Cooper Z Doll H: PN O’Connor M The natural course of bulimia nervosa and binge eating disorder in young women Arch Gen Psychiatry 2000 1 7 659 665 10.1001/archpsyc.57.7.659 10891036

[B5] Ciliska D Evaluation of two nondieting interventions for obese women West J Nurs Res 1998 1 1 119 135 10.1177/019394599802000108 9473971

[B6] Polivy J Herman CP Undieting: A program to help people stop dieting Int J Eat Disord 1992 1 3 261 268 10.1002/1098-108X(199204)11:3<261::AID-EAT2260110309>3.0.CO;2-F

[B7] Ernsberger P Koletsky RJ Biomedical rationale for a wellness approach to obesity: An alternative to a focus on weight loss J Soc Issues 1999 1 2 221 260 10.1111/0022-4537.00114

[B8] Wilfley DE Vannucci A White EK Early intervention of eating- and weight-related problems J Clin Psychol Med Settings 2010 1 4 285 300 10.1007/s10880-010-9209-0 20960039 PMC3705973

[B9] Flegal KM Graubard BI Williamson DF Gail MH Excess deaths associated with underweight, overweight, and obesity JAMA 2005 1 15 1861 1867 10.1001/jama.293.15.1861 15840860

[B10] Bacon L Aphramor L Weight science: Evaluating the evidence for a paradigm shift Nutr J 2011 1 9 2 13 21261939 10.1186/1475-2891-10-9 PMC3041737

[B11] Drapeau V Provencher V Lemieux S Després JP Bouchard C Tremblay A Do 6-y changes in eating behaviors predict changes in body weight? Results from the Quebec Family Study Int J Obes (Lond) 2003 1 7 808 814 10.1038/sj.ijo.0802303 12821966

[B12] Marchesini G Cuzzolaro M Mannuci E Dalle Grave R Gennaro M Tomasi F Barantani EG Melchionda N Weight cycling in treatment-seeking obese persons: data from the QUOVADIS study Int J Obes (Lond) 2004 1 11 1456 1462 10.1038/sj.ijo.0802741 15314631

[B13] Diaz CA Mainous AG III Everett CJ The association between weight fluctuation and mortality: Results from a population-based cohort study J Community Health 2005 1 3 153 165 10.1007/s10900-004-1955-1 15847242

[B14] Lissner L Odell PM D’Agostino RB Stokes J III Kreger BE Belanger AJ Brownell KD Variability of body weight and health outcomes in the Framingham population N Engl J Med 1991 1 26 1839 1844 10.1056/NEJM199106273242602 2041550

[B15] Petroni ML Vilanova N Aragnina S Fusco MAGF Compare A Marchesini G Psychological distress in morbid obesity in relation to weight history Obes Surg 2007 1 3 391 399 10.1007/s11695-007-9069-3 17546849

[B16] Carrier K Steinhardt M Bowman S Rethinking traditional weight management programs: A 3 year follow-up evaluation of a new approach J Psychol 1994 1 5 517 10.1080/00223980.1994.9914910 7983609

[B17] McFarlane T Polivy J McCabe RE Help, not harm: Psychological foundation for a non dieting approach toward health J Soc Issues 1999 1 2 261 276 10.1111/0022-4537.00115

[B18] Stice E A prospective test of the dual-pathway model of bulimic pathology: Mediating effects of dieting and negative affect J Abnorm Psychol 2001 1 1 124 135 11261386 10.1037//0021-843x.110.1.124

[B19] Polivy J Psychological consequences of food restriction J Am Diet Assoc 1996 1 6 589 592 10.1016/S0002-8223(96)00161-7 8655907

[B20] Yanovski SZ Billington CJ Epstein LH Goodwin NJ Hill JO Pi-Sunyer FX Rolls BJ Stern JS Wadden TA Weinsier RL Wilson GT Wing RR Van Hubbard S Hoofnagle JH Everhard J Harrison B Dieting and the development of eating disorders in overweight and obese adults Arch Intern Med 2000 1 17 2581 2589 10999971 10.1001/archinte.160.17.2581

[B21] Leon GR Fulkerson JA Perry CL Cudeck R Personality and behavioral vulnerabilities associated with risk status for eating disorders in adolescent girls J Abnorm Psychol 1993 1 3 438 444 8408956 10.1037//0021-843x.102.3.438

[B22] Telch CF Agras WS Obesity, binge eating and psychopathology. Are they related? Int J Eat Disord 1994 1 1 53 61 10.1002/1098-108X(199401)15:1<53::AID-EAT2260150107>3.0.CO;2-0 8124327

[B23] Foster G Wadden T Vogt R Brewer G What is a reasonable weight loss? Patient’s expectations and evaluations of obesity treatment outcomes J Consult Clin Psychol 1997 1 79 85 9103737 10.1037//0022-006x.65.1.79

[B24] Dalle Grave R Calugi S Molinari E Petroni ML Bondi M Compare A Marchesini G Group QS Weight loss expectations in obese patients and treatment attrition: an observational multicenter study Obes Res 2005 1 11 1961 1969 10.1038/oby.2005.241 16339128

[B25] National Health and Medical Research Council Clinical practice guidelines for the management of overweight and obesity in adults 2003 Melbourne, VIC: National Health and Medical Research Council

[B26] Miller WC How effective are traditional dietary and exercise interventions for weight loss? Med Sci Sports Exerc 1999 1 8 1129 1134 10.1097/00005768-199908000-00008 10449014

[B27] Wadden TA Crerand CE Brock JB Behavioral Treatment of Obesity Psychiatr Clin N Am 2005 1 151 170 10.1016/j.psc.2004.09.008 15733617

[B28] Reinehr T Widhalm K L’Allemand D Wiegand S Wabitsch M Holl RW Two-year follow-up in 21,784 overweight children and adolescents with lifestyle intervention Obesity 2009 1 6 1196 1199 19584877 10.1038/oby.2009.17

[B29] Wadden TA Sternberg JA Letizia KA Treatment of obesity by very low calorie diet, behavior therapy, and their combination. A five-year perspective Int J Obes (Lond) 1989 1 167 172 2613427

[B30] Guagnano MT VP-P Carrans C Merlittli D Sensi S Weight fluctuations could increase blood pressure in android obese women Clin Sci 1999 1 677 680 10.1042/CS19990050 10334976

[B31] Harrington M Gibson S Cottrell RC A review and meta-analysis of the effect of weight loss on all-cause mortality risk Nutr Res Rev 2009 1 1 93 108 10.1017/S0954422409990035 19555520

[B32] Pal S Egger G Wright G Dealing with obesity: An Australian perspective Asia Pac J Public Health 2003 1 33 36 10.1177/101053950301500S09 18924539

[B33] Miller WC The weight-loss-at-any-cost environment: How to thrive with a health-centred focus J Nutr Educ Behav 2005 1 SUPPL. 2 s89 s93 16246273 10.1016/s1499-4046(06)60205-4

[B34] Provencher V Begin C Tremblay A Mongeau L Boivin S Lemieux S Short-term effects of a “health at every size” approach on eating behaviors and appetite ratings Obesity 2007 1 4 957 966 10.1038/oby.2007.638 17426331

[B35] Robison J Weight, health and culture: Shifting the paradigm for alternative health care Complement Health Pract Rev 1999 1 1 45 69

[B36] Hawley G Horwath C Gray A Bradshaw A Katzer L Joyce J O’Brien S Sustainability of health and lifestyle improvements following a non-dieting randomised trial in overweight women Prev Med 2008 1 6 593 599 10.1016/j.ypmed.2008.08.008 18817809

[B37] Provencher V Begin C Tremblay A Mongeau L Corneau L Dodin S Boivin S Lemieux S Health-at-every-size and eating behaviors: 1-year follow-up results of a size acceptance intervention Am Diet Assoc 2009 1 1854 1861 10.1016/j.jada.2009.08.017 19857626

[B38] Bacon L Stern JS Van Loan MD Keim NL Size acceptance and intuitive eating improve health for obese, female chronic dieters J Am Diet Assoc 2005 1 6 929 936 10.1016/j.jada.2005.03.011 15942543

[B39] Rapaport L Clark M Wardle J Evaluation of a modified cognitive-behavioural programme for weight management Int J Obes (Lond) 2000 1 12 1726 1737 10.1038/sj.ijo.0801465 11126231

[B40] Goodrick GK Poston WSC II Kimball KT Reeves RS Foreyt JP Nondieting versus dieting treatment for overweight binge-eating women J Consult Clin Psychol 1998 1 2 363 368 9583339 10.1037//0022-006x.66.2.363

[B41] Tanco S Linden W Earle T Well-being and morbid obesity in women: a controlled therapy evaluation Int J Eat Disord 1998 1 3 325 339 10.1002/(SICI)1098-108X(199804)23:3<325::AID-EAT10>3.0.CO;2-X 9547667

[B42] Clarke GN Improving the transition from basic efficacy research to effectiveness studies: Methodological issues and procedures J Consult Clin Psychol 1995 1 5 718 725 7593864 10.1037//0022-006x.63.5.718

[B43] Fairburn CG Beglin SJ Assessment of eating disorders: Interview of self-report questionnaire? Int J Eat Disord 1994 1 4 363 370 7866415

[B44] Peterson CB Crosby RD Wonderlich SA Joiner T Crow SJ Mitchell JE Bardone-Cone AM Klein M Le Grange D Psychometric properties of the eating disorder examination-questionnaire: Factor structure and internal consistency Int J Eat Disord 2007 1 4 386 389 10.1002/eat.20373 17304585

[B45] Karlsson J Persson LO Sjostrom K Sullivan M Psychometric properties and factor structure of the Three-Factor Eating Questionnaire (TFEQ) in obese men and women. Results from the Swedish Obese Subjects (SOS) study Int J Obes (Lond) 2000 1 12 1715 1725 10.1038/sj.ijo.0801442 11126230

[B46] Stunkard AJ Messick S The three-factor eating questionnaire to measure dietary restraint, disinhibition and hunger J Psychosom Res 1985 1 1 71 83 10.1016/0022-3999(85)90010-8 3981480

[B47] Lohse B Satter E Horacek T Gebereslassi T Oakland MJ Measuring Eating Competence: Psychometric Properties and Validity of the ecScatter Inventory J Nutr Educ Behav 2007 1 5 141 198 10.1016/j.jneb.2007.04.371 17826696

[B48] Stotts JL Lohse B Reliability of the ecSatter Inventory as a tool to measure eating competence J Nutr Educ Behav 2007 1 5 SUPPL s167 s170 17826697 10.1016/j.jneb.2007.03.091

[B49] Cash TF MBSRQ User’s Manual 2000 3 Norfolk, VA: Old Dominion Univer. Press

[B50] Cooper PJ Tayor MJ Cooper Z Fairburn CG The development and validation of the Body Shape Questionnaire Int J Eat Disord 1987 1 4 485 494 10.1002/1098-108X(198707)6:4<485::AID-EAT2260060405>3.0.CO;2-O

[B51] Rosen JC Jones A Ramirez E Waxman S Body Shape Questionnaire: Studies of validity and reliability Int J Eat Disord 1996 1 3 315 319 10.1002/(SICI)1098-108X(199611)20:3<315::AID-EAT11>3.0.CO;2-Z 8912044

[B52] Lovibond PF Lovibond SH Manual for the depression, anxiety, and stress scales 1995 Sydney: Psychology Foundation

[B53] Crawford JR Henry JD The Depression Anxiety Stress Scale (DASS): Normative data and latent structure in a large non-clinical sample Br J Clin Psychol 2003 1 111 131 10.1348/014466503321903544 12828802

[B54] Markland DA Ingledew DK The measurement of exercise motives: Factorial validity and invariance across gender of a revised Exercise Motivations Inventory Br J Health Psychol 1997 1 4 361 376 10.1111/j.2044-8287.1997.tb00549.x

[B55] Gollings EK Paxton SJ Comparison of internet and face-to-face delivery of a group body image and disordered eating intervention for women: A pilot study Eat Disord: J Treat Prev 2006 1 1 15 10.1080/10640260500403790 16757445

[B56] Paxton SJ McLean SA Gollings EK Faulkner C Wertheim EH Comparison of face-to-face and internet interventions for body image and eating problems in adult women: an RCT Int J Eat Disord 2007 1 692 704 10.1002/eat.20446 17702020

[B57] McLean SA Paxton SJ Wertheim EH A body image and disordered eating intervention for midlife women: A randomised controlled trial J Consult Clin Psychol 2011 1 6 751 758 22004306 10.1037/a0026094

[B58] Cohen JW Statistical power analysis for the behavioral sciences 2nd ed 1988 Hillsdale, NJ: Lawrence Erlbaum Associates

[B59] Wolk SL Loeb KL Walsh BT Assessment of patients with anorexia nervosa: Interview versus self-report Int J Eat Disord 2005 1 92 99 10.1002/eat.20076 15732073

[B60] de Lauzon B Romon M Deschamps V Lafay L Borys J Karlsson J Ducimetiere P Charles MA Group FLVSS The three-factor eating questionnaire-R18 is able to distinguish among different eating patterns in a general population J Nutr 2004 1 2372 2380 15333731 10.1093/jn/134.9.2372

[B61] Moor S Vartanian LR Touyz SW Beuamont PJV Psychopathology of EDNOS patients: To whom do they compare? Clin Psychol 2004 1 2 70 75 10.1080/1328420041233130436

[B62] Friedman MA Brownell KD Psychological Correlates of Obesity: Moving to the next research generation Psychol Bull 1995 1 1 3 20 7870862 10.1037/0033-2909.117.1.3

[B63] Hrabosky JI Thomas JJ Elucidating the relationship between obesity and depression: Recommendations for future research Clin Psychol Sci Pract 2008 1 1 28 34 10.1111/j.1468-2850.2008.00108.x

[B64] Moroshko I Brennan L O’Brien P Predictors of dropout in weight loss interventions: a systematic review of the literature Obes Rev 2011 1 912 934 10.1111/j.1467-789X.2011.00915.x 21815990

[B65] Nauta H Hospers H Jansen A One-year follow-up effects of two obesity treatments on psychological well-being and weight Br J Health Psychol 2001 1 3 271 284 10.1348/135910701169205 14596727

[B66] Crerand CE Ta W Foster GD Sarwer DB Paster LM Berkowitz RI Changes in obesity-related attitudes in women seeking weight reduction Obesity 2007 1 740 747 10.1038/oby.2007.590 17372325

[B67] Jackson EG Eating Order: A 13-Week Trust Model Class for Dieting Casualties J Nutr Educ Behav 2008 1 1 43 48 10.1016/j.jneb.2007.01.007 18174104

